# Glaucoma and Antioxidants: Review and Update

**DOI:** 10.3390/antiox9111031

**Published:** 2020-10-22

**Authors:** Jose Javier Garcia-Medina, Elena Rubio-Velazquez, Maria Dolores Lopez-Bernal, Alejandro Cobo-Martinez, Vicente Zanon-Moreno, Maria Dolores Pinazo-Duran, Monica del-Rio-Vellosillo

**Affiliations:** 1 Department of Ophthalmology, General University Hospital Morales Meseguer, 30008 Murcia, Spain; elena.rubio@carm.es (E.R.-V.); dolores.lopez11@carm.es (M.D.L.-B.); alexcobo96@hotmail.com (A.C.-M.); 2 Department of Ophthalmology and Optometry, University of Murcia, 30120 Murcia, Spain; 3 Ophthalmic Research Unit Santiago Grisolia, 46017 Valencia, Spain; vczanon@universidadviu.com (V.Z.-M.); dolores.pinazo@uv.es (M.D.P.-D.); 4 Red Temática de Investigación Cooperativa en Patología Ocular (OFTARED), Instituto de Salud Carlos III, 28029 Madrid, Spain; 5 Area of Health Sciences, Valencian International University, 46002 Valencia, Spain; 6 Department of Ophthalmology, University of Valencia, 46010 Valencia, Spain; 7 Department of Anesthesiology, University Hospital Virgen de la Arrixaca, 30120 Murcia, Spain; monicadelriov@hotmail.com

**Keywords:** glaucoma, antioxidant, oxidative stress

## Abstract

Glaucoma is a neurodegenerative disease characterised by the progressive degeneration of retinal ganglion cells. Oxidative stress has been related to the cell death in this disease. Theoretically, this deleterious consequence can be reduced by antioxidants substances. The aim of this review is to assemble the studies published in relation to antioxidant supplementation and its effects on glaucoma and to offer the reader an update on this field. With this purpose, we have included studies in animal models of glaucoma and clinical trials. Although there are variable results, supplementation with antioxidants in glaucoma may be a promising therapy in glaucoma.

## 1. Introduction

Glaucoma is a neurodegenerative disease characterised by slow and progressive apoptosis of retinal ganglion cells (RGCs) that affects more than 70 million people worldwide, making it one of the leading causes of irreversible blindness. Patients suffer from a centripetal loss of visual field associated with an increasing optic disc cupping remaining asymptomatic until reaching advanced stages, which means that the number of affected individuals is much greater than those who know to have it. The most common type of glaucoma affecting population is primary open-angle glaucoma (POAG), but there are other types such as primary angle closure glaucoma or secondary glaucomas due to another disturbance.

Some risk factors to develop POAG are myopia, advanced age, female sex or family history of glaucoma but only one of them, chronic elevation of intraocular pressure (IOP), is modifiable. IOP is the result of a fine balance between the production of aqueous humour inside the eye and its drainage outside. The main cause of IOP increase is the difficulty of aqueous humour outflow at the trabecular meshwork, a structure located in the iridocorneal angle.

High IOP is believed to alter the homeostasis of the retina and optic nerve in glaucoma through mechanical damage and hypoperfusion. Therefore, the treatment of this disorder currently aims to decrease IOP by medical and surgical means. But there are glaucomatous patients that present progression even with IOP under control. Plus, normotensive glaucoma, a kind of glaucoma with normal IOP, also progresses with IOP in normal ranges. Immunological components and toxic reactions have also been studied in glaucoma. Thus, pathophysiology of this entity is complex and not completely known [1].

Oxidative stress has been considered to play a role in the development of glaucoma [2], not only in the eye of glaucoma patients [3] but also in plasma. In fact, it has been shown that total antioxidant status (TAS) is decreased in glaucoma [4,5]. Furthermore TAS is directly correlated to increased optic disc cupping in POAG [4] and inversely to IOP in PACG [5].

Otherwise, oxidative stress may affect the RGCs and other retinal cells in the posterior segment of the eye [6] but also to the trabecular meshwork in the anterior segment [6,7]. These two phenomena have been shown to occur early in the development of glaucoma [8,9].

In this context, antioxidant supplementation has been considered as a possible therapy against glaucoma, especially when irreversible damage of trabecular meshwork or RGCs has not appeared yet.

Pre-clinical studies for assessing antioxidant supplementation in animal models of glaucoma have been performed. The majority of these have been conducted in rodents as can be seen below. The murine model has important similarities with human eye in the study of glaucoma such as aqueous humour dynamics and optic disc cupping in response to high IOP, which is easily achieved in rats or mice. Furthermore, it is a low-cost model, easy to be manipulated genetically and relatively easy for varying the diet or obtaining the eyes. However, in this context other animal models have also been considered. Animal model of glaucoma have been extensively described elsewhere [10,11].

A number of studies have also been performed in glaucomatous patients administering antioxidants as therapeutical agents. The aim of this review is to assemble updated information about animal models of glaucoma and clinical trials dealing with antioxidant supplementation in glaucoma.

## 2. Antioxidant Supplementation in Animal Models of Glaucoma

Several studies have been performed using antioxidant supplementation in animal models of glaucoma. They are summarised in Table 1.

A wide variety of studies have been performed in different animal models using several antioxidants in an attempt to clarify the role that these substances may play in the pathophysiology of glaucoma.

Vitamin A is a group of natural fat-soluble unsaturated nutritional organic compound that includes retinol, retinal, and several provitamin A carotenoids. Its possible antioxidant effect was studied by Das et al. [12]. They evaluated lipid peroxidation (induced by ascorbic acid or iron) using rat brain homogenates. They found that increasing concentrations of several vitamin A analogues reduced lipid peroxidation in a dose-dependent manner.

Vitamin E is another natural fat-soluble antioxidant that includes tocotrienol and tocopherol. Ko et al. [22] studied the effect of a vitamin E-deficient diet in a model of surgically induced glaucoma in rats (by cauterizing three episcleral veins of the left eye of each rat). They found that Vitamin E deficiency increased the retinal ganglion cell loss produced by glaucoma, as well as lipid peroxidation in the retinas of rats with hypertensive eyes. These findings suggest once again the relevance of antioxidants in the diet. A different and interesting approach was observed in the study by Hsu et al. [29]. They used beagle dogs suffering from open angle glaucoma. Contact lenses were loaded with dorzolamide and timolol, observing that these contact lenses were more effective in lowering intraocular pressure than eye drops. In addition, coloading the lenses with vitamin E increased the antihypertensive effect and also the duration of timolol + dorzolamide treatment. These findings would help in the topical treatment of glaucoma in humans, although deep study in the subject would be needed.

B vitamins are a class of natural water-soluble vitamins. Williams et al. [31] used DBA/2J mice, suffering from genetic, age-related glaucoma. They reported that vitamin B3 supplementation protected against retinal ganglion cell damage and mitochondrial degeneration and also against optic nerve fibres loss. Vitamin B3 was also used in a study performed by Chou et al. [39] in DBA/2J mice and found that this antioxidant protected retinal ganglion cell function (estimated by the effect of flicker on electroretinogram) and also retinal ganglion cells density and mitochondria. These findings agree with the ones by Williams et al. [31].

Vitamin C, also known as ascorbic acid and ascorbate, a natural water-soluble vitamin was used by Xu et al. [26] to evaluate its effects on porcine trabecular meshwork cells. They reported antioxidant effects associated with induction of autophagy and lysosomal proteolysis. Based on these findings, they hypothesised that vitamin C might protect the outflow pathway cells from age-related degradation. Therefore vitamin C would also produce a beneficial effect against glaucoma.

Coenzyme Q, also known as ubiquinone, is a natural coenzyme family that is ubiquitous in animals and most bacteria. Nucci et al. [17] used a model of retinal ischemia-reperfusion in rats by raising intraocular pressure above arterial pressure. In order to achieve this the anterior chamber of the right eye was cannulated with a 27-gauge infusion needle connected to a 500-ml plastic container of sterile saline, the IOP was raised to 120 mmHg for 45 min by elevating the saline reservoir. Retinal ischemia was confirmed by observing whitening of the iris and loss of the red reflex of the retina. They found that intravitreal administration of coenzyme Q10 reduced the glutamate increase seen after ischemia, and that also protected the retinal ganglion cells from apoptosis. Nakajima et al. [18] demonstrated in cultured retinal ganglion cells that the coadministration of coenzyme Q10 and a soluble vitamin E derivative protected from cellular damage and apoptosis induced by hydrogen peroxide or N-methyl-d-aspartate (NMDA). These findings, among others, were reviewed by Russo et al. [19] in their model of retinal ischemia induced by high IOP. They concluded that the protective effects seen on retinal ganglion cell apoptosis after ischemia during treatment with NMDA glutamate receptor antagonists, nitric oxide synthase inhibitors, or coenzyme Q10 support an important role for excitotoxicity in the mechanisms underlying retinal ganglion cell death. Lee et al. [25] used glaucomatous DBA/2J mice. In this case the animals were given a diet supplemented with coenzyme Q10. They reported an improvement in retinal ganglion cell survival and preservation of the axons in the optic nerve head by increasing the expression of superoxide dismutase 2 and heme oxygenase 1 proteins, among others in the supplemented group, compared to control. Davis et al. [32] used a surgically induced model of glaucoma in rats by injecting a solution of saline in episcleral veins of one eye. They found that the topical administration of coenzyme Q10 reduced retinal cell apoptosis and produced a significant retinal neuroprotective effect. They also performed in vitro experiments with murine retinal mixed cultures and an immortalised retinal neuronal cell line. The results were similar to the ones observed in the in vivo study.

Lipoic acid (LA), also known as α-lipoic acid and alpha lipoic acid (ALA) and thioctic acid, is a natural organosulfur compound derived from caprylic acid (octanoic acid). Chidlow et al. [13] used a rat model with induced ischemia by cannulating the anterior chamber of one eye with a 30-gauge needle connected to an elevated reservoir of sterile balanced salt solution. This generated an intraocular pressure of more than 120 mmHg that was maintained for 45 minutes before allowing reperfusion to commence. The rats were then treated chronically with intraperitoneal α-lipoic acid. They reported a protective effect of retinal ganglion cells from ischemic damage. This effect was confirmed in retinal cells in culture. α-lipoic acid seemed to protect GABA-immunoreactive neurons from anoxia. Inman et al. [24] gave α-lipoic acid, to glaucomatous DBA/2J mice in the diet. Their study showed increased antioxidant gene and protein expression, increased protection of retinal ganglion cells and improved retrograde axonal transport compared to controls.

Extract from the tree Ginkgo biloba (commonly known as ginkgo or gingko, also known as the maidenhair tree) was also tested as antioxidant. Hirokaa et al. [14] used rats with surgically induced unilateral chronic glaucoma. Moderately elevated IOP was produced by cautery of three episcleral vessels. Animals were fed ad libitum with a ginkgo biloba extract and maintained in temperature-controlled rooms. They reported a reduction in retinal ganglion cell loss when compared with their contralateral control eyes with normal IOP. The same antioxidant was tested by Eckert [15] and coworkers. They reviewed the protective effects of a ginkgo biloba extract on PC12 cells in culture. The PC12 cells suffer from mutations that make them more susceptible to oxidative stress and mitochondrial dysfunction and are used as an in vitro model simulating the effects of Alzheimer’s disease. These cells were subjected to a variety of aggressions, including hydrogen peroxide attack, and respiratory chain inhibitors. The ginkgo biloba extract protected mitochondrial function and prevented apoptosis. These beneficial effects were confirmed in dissociated brain cells and isolated brain mitochondria from mice against oxidative stress produced by hydrogen peroxide. Cybulska-Heinrich et al. [23] reviewed the anti-inflammatory effects, the mitochondrial protection, and also the antithrombotic and vascular beneficial effects attributed to ginkgo biloba. This review was focused on the mechanisms mediating the protective effects of ginkgo biloba in several animal models of glaucoma, hypothesizing that they are mainly due to its antioxidant properties and to mitochondrial function protection. Finally, the authors try to make sense of the beneficial effects of this plant extracts on human glaucoma patients, including reduced visual field damage, but the available data do not allow for conclusions about the mechanisms involved to be drawn.

Omega 3 and omega 6 are natural polyunsaturated fatty acids found in animals and vegetables. Nguyen et al. [16] measured intraocular pressure and aqueous humour outflow in rats given an omega 3-deficient diet, and found increased IOP and reduced outflow, compared with rats with normal dietary omega 3 fatty acids. Schnebelen et al. [21] gave a diet enriched in omega 3 and 6 fatty acids to rats with surgically induced glaucoma. IOP elevation was induced in the right eye by photocoagulation of the episcleral veins, limbus and trabecular meshwork using a 532 nm green laser. Animals treated with the enriched diet did not show a stop in the rise of intraocular pressure, but a lowered glial activation was observed. This allowed to prevent retinal degenerative changes produced by increased IOP.

Resveratrol is a stilbenoid, a type of natural phenol, and a phytoalexin produced by several plants in response to injury or when the plant is under attack by pathogens, such as bacteria or fungi. Pirhan et al. [30] in their study used resveratrol and a neuroprotective drug riluzol. They administered both separately or in combination to rats with glaucoma induced in one eye of each animal by intracameral injection of hyaluronic acid. They found that both treatments, alone or in combination, reduced the retinal ganglion cell loss produced in this model of elevated intraocular pressure. This reduction was higher in the combination treated group. Luo et al. [33] used a model of retinal ischemia induced by increased intraocular pressure. Resveratrol reduced retinal damage and ganglion cell loss. These beneficial effects were associated with attenuated retinal apoptosis, reactive gliosis, and inflammation. This compound was also used in a study performed by Zhang et al. [34]. They evaluated the effects of this antioxidant in a rat model of glaucoma induced by injecting micro-magnetic iron beads into the anterior chamber, and in RGC-5 cells in culture (transformed retinal ganglion cells) exposed to increased hydrostatic pressure. Both models showed apoptosis and mitochondrial dysfunction after hypertension that were prevented by resveratrol, associated with a reduction in radical oxygen species generation. The antioxidative effect of resveratrol was also studied by Cao et al. [38]. They evaluated the effects of this antioxidant in a murine model of glaucoma induced by injecting polystyrene microbeads into the anterior chamber, as well as in murine retinal ganglion cells in culture. Resveratrol seemed to protect retinal ganglion cells from degeneration (with no effects on intraocular pressure) and reduced retinal reactive oxygen species. This was associated with changes in the expression of multiple proteins involved in cellular stress response, oxidative stress and apoptosis. Once again positive effects of this antioxidant are observed, in concordance with the results obtained in previous studies ([30,33,34]).

α-Luminol an antioxidant and anti-inflammatory drug was tested by Gionfriddo et al. [20]. They used this drug on glaucomatous DBA/2J mice, which suffer from oxidative stress, reduced retinal glutathione levels and also reduced glutamate and glutamine synthase neuronal levels. Treatment with α-Luminol prevented those changes. These results are consistent with the hypothesis that oxidative stress contributes to the retinal changes in glaucoma.

Can et al. [27] investigated the effects of ghrelin (a hormone with antioxidant properties) in a model of surgically induced glaucoma in rats by cauterizing limbal veins. Glaucoma elevated the levels of malondialdehyde, nitric oxide and nitric oxide synthase-2 in anterior chamber fluid, indicating oxidative and nitrosative stress, associated with apoptosis and increased expression of apoptosis-related proteins. Treatment with ghrelin prevented those changes. These finding suggest an important role for oxidative stress in the pathogenesis of the disease.

SkQ1, a mitochondria-targeted antioxidant approved for therapy of polyethiological dry eye disease was used by Iomdina et al. [28] in a model of glaucoma induced by injecting hydroxypropylmethylcellulose into the anterior chamber of rabbits. The authors demonstrated that SkQ1 reduced intraocular pressure and protected optical nerve axons from degeneration in this model of experimental glaucoma.

The antioxidative effect of green tea extract has also been used in a study by Yang et al. [35]. They evaluated this antioxidative effect on the retinal ganglion cell loss produced by retinal ischemia-reperfusion induced by raisin intraocular pressure up to 110 mmHg. Exposure to the extract reduced ganglion cell loss and apoptosis, as well as the increased expression of inflammation and apoptosis-related proteins. These finding suggest an important role for oxidative stress in the pathophysiology of this response.

Wu et al. [36] investigated the neuroprotective effects of the mitochondria-targeted antioxidant Szeto-Schiller peptide 31 (SS-31) in a rat experimental glaucoma model, induced by injecting polystyrene microspheres into the anterior chamber. They observed that SS31 ameliorated the alterations in electroretinogram produced by glaucoma, reduced oxidative stress and ganglion cell loss and mitochondrial-mediated apoptosis.

Angel Aillegas et al. [37] studied the antioxidant effect of synthesised ascorbyl laurate (ASC12)-based nanostructures applied topically to the cornea in a glaucoma model performed in rabbits by intracameral injections of alpha-chymotrypsin in the right eye. Treatment with ASC12 increased the antioxidant capacity of the aqueous humour in hypertensive rabbits.

Tanaka-Gonome et al. [40] evaluated the effects of Astaxanthin (AST), a natural marine carotenoid, on the glutamate/aspartate transporter (GLAST)-deficient (GLAST^-/-^) mouse, a model of normal tension glaucoma (NTG), caused by both the glutamate neurotoxicity and oxidative stress in the retina. AST significantly attenuated the thinning of the ganglion cell complex in GLAST^-/-^ mice in comparison to controls.

Although the above results are promising they have to be taken with caution. The studies have been performed in animal models and cannot be extrapolated to human therapy.

## 3. Antioxidant Supplementation in Clinical Trials in Glaucoma

Several studies carried out in recent years have shown that antioxidants induce a beneficial effect on patients with glaucoma (Table 2).

Omega-3 fatty acids play a fundamental role in cellular metabolism, acting as a protector at the cellular level. Glaucoma patients have reduced levels of omega-3 fatty acids. Several studies about supplementation with omega-3 fatty acids have yielded mixed results. In 1990 a study demonstrated the efficacy of polyunsaturated fatty omega-3 acids in patients with glaucoma, showing an improvement in the blue/yellow perimetry indices. It revealed a decrement in MD after 3 months of oral supplementation [41]. In contrast, a more recent study comparing antioxidant supplementation with and without omega-3 fatty acids found no statistically significant differences between groups, with similar deterioration of MD in all patients [53].

Ginko biloba is a medicinal plant from China. It has been used for more than 2000 years. It is an antioxidant that has previously been shown to have a beneficial effect on blood circulation due to its vasoregulatory and rheological effect. Other known effects are the inhibition of nitric oxide as well as a neuroprotective activity [42]. These effects can be beneficial in patients with normal-tension glaucoma, based on vascular theory of mechanisms of glaucomatous optic nerve damage. Quaranta et al. verified that supplementation with Ginko Biloba in patients with normal-tension glaucoma could improve the already existing visual field damage in these patients. They achieved an MD reduction of 11.40 + 3.27 decibels (dB) compared to the 8.78 + 2.56 dB reduction in patients treated with placebo (*p* = 0.0001) [42].

Shim obtained similar results using a combination of Ginko Biloba and anthocyanins from *Vaccinium Myrtillus* and bilberry. In this study, the authors not only achieved a significant improvement in HVF parameters of the visual field. They also reported a significant improvement in best corrected visual acuity (BCVA) after two years of oral supplementation [45].

Ginko biloba, in addition to preventing cell damage at the membrane level, can improve blood circulation, and may be beneficial in improving circulation at the optic nerve level. Administration of ginko biloba extract (GBE) by oral supplementation may also have beneficial effects on peripapillary circulation. Park et al. used the Heidelberg Retina Flowmeter (HRF. Heidelberg engineering, Heidelberg, Germany) to measure blood flow. They obtained statistically significant improvements in blood volume (in the upper quadrants) and blood velocity (in the temporal areas of the neuroretinal ring, both upper and lower peripapillary area) [44].

A recently published study [55] supports these results. In a similar way to that mentioned above, the retinal perfusion of the capillary beds was measured by the Heidelberg Retinal Flowmeter (HRF). In addition, flow velocity and vascular resistance was measured at the level of the ophthalmic artery, central retinal artery and posterior temporal ciliary artery. The patients were given a dietary supplement composed of vitamins, minerals, omega-3 fatty acids and botanical extracts, including Ginko Biloba. An increase in the speed of retroocular blood flow and also a decrease in vascular resistance in the central retinal artery as well as in the posterior short nasal cyclic arteries were observed after one month of oral supplementation. An improved blood flow at the retinal level was also detected.

These satisfactory results have not been demonstrated in cases of normal-tension glaucoma in its earliest stages. In this context, Guo et al. studied initial cases of normal stress glaucoma. They assessed the effect on the visual field and contrast sensitivity. They were unable to demonstrate any improvement in the patients treated [52].

Abnormal levels of plasma endothelin-1 are detected in glaucoma patients [48]. Anthocyanins, a polyphenol found in red wine, can be beneficial to patients with primary open-angle glaucoma. Anthocyanins are a flavonoid that have a powerful antioxidant effect. At a vascular level it is essential because it reduces capillary fragility and inhibits platelet aggregation. Ohguro et al. found a significant improvement in eye circulation in patients taking this antioxidant compared to the placebo group. The exact mechanism involved in this improvement is not known, although normalisation of plasma levels of endothelin-1 appears to be involved. Despite the improvement in ocular circulation, no changes in intraocular pressure were detected in this study [44]. But similar studies with black currant anthocyanins showed a significant improvement in intraocular pressure compared to placebo in both healthy and glaucoma patients. In glaucoma patients, furthermore, the deterioration of MD over the two-year follow-up period was less in patients who received oral supplementation [48].

Oral supplementation with essential polyunsaturated fatty acids associated with antioxidants (Brudysec^®^) achieved a reduction in oxidative stress markers (interleukin-3, interleulin-6, vascular endothelial grow factor, tumour necrosis factor α) and a significant improvement in dry eye symptoms present in glaucoma patients such as eye heaviness, burning or photophobia after 3 months of treatment [48]. Oral administration of a multivitamin supplement (NuaDHA Vision^®^ + DHA1000^®^) containing, among others, R-alpha lipoic acid (ALA), a short-chain fatty acid with anti-inflammatory and antioxidant activity, produces a reduction in prooxidative activity, which is reflected in a decrease in the peripheral blood values of malondialdehyde (MDA) and an increase in the total antioxidant status (TAS), these changes being greater in patients with glaucoma than in healthy patients. They also reported an improvement in the dry eye symptoms typically experienced by patients treated for glaucoma, both subjectively and objectively, with treated patients showing a significantly better Schirmer test than untreated patients [59].

Increased oxidative stress and inadequate antioxidant defence may be involved in the pathogenesis of the neurological process in patients with pseudoexfoliative glaucoma. Docosahexanoic acid (DHA) is a well-known fatty acid present in olive oil and blue fish. Other less known foods can also be a source of omega-3, such as avocado, spinach or nuts such as walnuts, and seeds, such as chia or flaxseed. DHA can be useful in the physiopathological development of pseudoexfoliative glaucoma (PSX), ameliorating the subclinical inflammation that these patients present. After 6 months of oral supplementation with this compound, there was a reduction in oxidative stress. This improvement was reflected in a decrease of IL-6 and malondialdehyde in plasma (MDA [56]).

Falsini et al. studied the effect of epigallocatechin-gallate (EGC), a powerful antioxidant present in remarkable quantities in green tea. Although they could not demonstrate an improvement or stabilisation in the visual field, they performed pattern-evoked electroretinograms (PERGs) on patients with ocular hypertension and primary open-angle glaucoma, with the aim of assessing the functionality of the inner retina. These patients were given an EGCG supplement for three months. In the glaucoma patients, a modest improvement in the amplitude of the PERGs was observed [43].

A promising pathway for the use of antioxidants in ocular pathology is the topical route. Coqun^®^ is a topical treatment of coenzyme Q-10 and vitamin E. Ozates et al. administered two drops of Coqun^®^ a day and measured markers of oxidative stress at the level of aqueous humour (superoxide dismutase and malondialdehyde) in patients with pseudoexfoliative glaucoma. After one month of treatment they found a statistically significant reduction of superoxide dismutase, but not significant changes in malondialdehyde level were observed after the 1-month follow-up period. [57].

The combination of various antioxidants can have a synergistic effect and provide a neuroprotective effect that ameliorates damage at the ganglion cell level in patients with glaucoma. Forksolin is an extract used mainly in traditional Chinese medicine. It is a labdanum diterpene produced by the indigenous plant Coleus (Plectranthus barbatus). Mutolo et al. studied the effect of an oral supplement containing forksolin, homotaurine, fleshy, folic acid, magnesium and vitamins B1, B2 and B6 in patients with open-angle glaucoma. They showed promising results after 12 months of treatment, with a reduction in intraocular pressure (mainly due to forksolin) and an improvement in the electroretinogram (PERG) pattern [54].

Saffron is one of the most ancient crops of mankind already used by the Egyptians, and mentioned by the texts of Hippocrates and Galen. It is a spice obtained from the stigmas of the Crocus sativus flower. Saffron, a species widely used in traditional cooking, can have beneficial effects on glaucoma. Saffron contains carotenoid derivatives such as crocin and crociten, with stronger antioxidant power. A dose of 30 mg/day orally succeeded in reducing intraocular pressure at one month of treatment in patients with open-angle glaucoma compared to patients treated with placebo [51].

Despite the promising results of the above-mentioned studies, they should be assessed with caution. The small number of patients included and the diversity of protocols make it difficult to generalise the results. Scuteri et al. have recently conducted a systematic review of the published literature. Of the 1615 papers included in the first search, only 6 met the criteria of the PRISMA flow chart [60]. Therefore, because of the great heterogeneity of the published works, the evidence to generalise the results is inconclusive. Studies with an adequate design and methodology and an adequate statistical power are needed.

## 4. Discussion and Conclusions

Every year, glaucoma causes loss of vision in thousands of people. Despite the fact that there are numerous treatments and surgical approaches, it is not possible to reverse its evolution in many cases. Therefore, there are a number of studies investigating new types of treatments that can be effective in the fight against this disease. One of these therapeutical lines is antioxidant supplementation.

A wide variety of studies have shown that antioxidant may help to regulate IOP as well as to protect RGCs against oxidative stress in glaucomatous animal models. However, although some studies showed promising results, human trials have not shown clearly any effective antioxidant formulation to be used in glaucoma treatment [60]. We should consider that clinical trials are not comparable to investigations performed in laboratory animals in which diet and the rest of living habits are under tight control. In contrast, in clinical trials investigators are able to control a part of participants’ behaviour. Additionally, metabolism and aging of animals and supplemented doses of antioxidants differ from the human being context [10,11,61].

In recent studies, there is a trend to combine together several antioxidants. In fact, synergetic effects of antioxidants have been proved when supplemented in combination [62].

This strategy has pros and cons. On the one hand, multiple antioxidants may act against multiple targets and, theoretically, it could present more beneficial effects. On the other hand, it is difficult to find out the exact effect of each antioxidant when combined. Plus, there is a chance of positive or negative interaction of different antioxidants among them with uncertain effects.

Otherwise, to make things more complex, antioxidants have been recently shown to act not only directly through its well-known electron-donating activity but also by indirect effects over the expression and action of different proteins, activating specific cellular receptors or modulating enzymatic effectors. One of this new mechanisms of action of antioxidants, called hormesis, is related to the upregulation of enzymes that act on detoxification processes or the overexpression of genes associated with repairing pathways [63].

Another important concern is to define the optimal doses of antioxidant supplementations. It is hard to define this aspect considering the other two major factors: the safety of the antioxidant or combination of antioxidants [64] and the duration for many years of human glaucoma.

Further studies have to be implemented to test long-term efficacy and safety of different combinations of antioxidants in order to find a useful formulation against a sight-threatening, lifelong disease like glaucoma.

## Figures and Tables

**Table 1:** Summary of antioxidant supplementation in animal studies (chronological order).

Year of Publication	Authors [Reference]	Investigated Animals	Used Antioxidant	Results
1989	Das et al. [12]	Wistar rat.	Vitamin A (retinol)	Inhibition of lipid peroxidation in rat brain homogenates.
2002	Chidlow et al. [13]	Wistar rat.	*α-*lipoic acid	Reduction of ganglion cell death after ischemia.
2004	Hirokaa et al. [14]	Sprague-Dawley rat.	Ginkgo biloba	Reduction in retinal ganglion cell loss in eyes with chronic, moderately elevated IOP.
2005	Eckert [15]	Review: a variety of cell cultures from different sources.	Ginkgo biloba	Stabilisation and protection of mitochondrial function of TC12 cells in culture.
2006	Nguyen et al. [16]	Sprague-Dawley rat.	Omega 3	Decrease in IOP associated with a significant increase in outflow facility and a decrease in ocular rigidity.
2007	Nucci et al. [17]	Wistar rat.	Coenzyme Q10	Reduction of glutamate and of retinal ganglion cell apoptosis after ischemia-reperfusion.
2008	Nakajima et al. [18]	RGC-5, (a rat ganglion cell-line transformed using E1A virus) rat.	Coenzyme Q10 Troxol (soluble vitamin E derivative)	Reduction of retinal ganglion cell (in culture) damage and apoptosis induced by hydrogen peroxide and NMDA.
2008	Russo et al. [19]	Rat (unspecified strain).	Coenzyme Q10 NMDA receptor antagonists Nitric oxide synthesis inhibitors	Review: Coenzyme Q10, NMDA receptor antagonists and nitric oxide synthesis inhibitors afford retinal protection supporting an important role for excitotoxicity in the mechanisms underlying retinal ganglion cell death.
2009	Gionfriddo et al. [20]	DBA/2J mice.	Alfa-luminol	Prevention of decrease in glutamate, glutathione and glutamine synthetase.
2009	Schnebelen et al. [21]	Sprage-Dawley rat	Omega-3 Omega-6	A supplementation of combined fatty acids is more effective than individual supplementation in preventing retinal damage due to increased intraocular pressure.
2010	Ko et al. [22]	Wistar rat.	Vitamin E	Increase in lipid peroxidation and elevated concentration of glutathione in vitamin E deficient rats compared to controls.
2012	Cybulska-Heinrich et al. [23]	Rat (unspecified strain) and human.	Ginkho Biloba	Review about the mechanisms mediating the beneficial effects on animal models of glaucoma and also in human patients.
2013	Inman et al. [24]	DBA/2J mice.	α-lipoic acid (ALA)	Increase antioxidant gene and protein expression, increase protection of RGCs and improved retrograde transport in treated subjects compared to control.
2014	Lee et al. [25]	DBA/2J mice.	Coenzyme Q10	Promotion of retinal ganglion cell survival and preservation of axons in the optic nerve head in glaucomatous DBA/2J mice.
2014	Xu et al. [26]	Porcine TM cells in culture.	Vitamin C (Ascorbic Acid)	Antioxidant properties with lower ferritin and basal iROS (intracellular reactive oxygen species) content, induction of autophagy, as well as increased lysosomal proteolysis in trabecular meshwork cells.
2015	Can et al. [27]	Sprague-Dawley rat.	Ghrelin	Decrease in apoptosis and related proteins: expression of GFAP (glial fibrillary acidic protein), S-100, and vimentin compared to control group. Reduction of oxidative and nitrosative stress.
2015	Iomdina et al. [28]	Male pigmented rabbit.	10-(6′-plastoquinonyl) decyltriphenylphosphonium (SkQ1)	Reduction of IOP and preservation of optical nerve axons.
2015	Hsu et al. [29]	Beagle dog.	Vitamin E	Greater and longer lasting decrease of IOP using contact lenses coloaded with timolol, dorzolamide and vitamin E compared with timolol and dorzolamide-loaded lenses or eye drops.
2016	Pirhan et al. [30]	Wistar rat.	Riluzol and Resveratrol	Preservation of retinal ganglion cells in all treated groups of rats with chronic elevation of IOP induced by intracameral injection of hyaluronic acid.
2017	Williams et al. [31]	DBA/2J mice.	Vitamin B3	Protection prophylactically and as an intervention against mitochondrial vulnerability and optic nerve degeneration.
2017	Davis et al. [32]	Primary murine (C57BL/6) retinal mixed cultures (pMC) and an immortalised retinal neuronal (RN) cell line (RGC5). Adult male Dark Agouti (DA) rats.	Coenzyme Q10	Reduced detection of apoptotic retinal cells in both in vitro and in vivo-treated groups when compared to control.
2018	Luo et al. [33]	Sprague-Dawley rat.	Resveratrol	Reduced retinal damage and retinal ganglion cell loss after retinal ischemia-reperfusion.
2018	Zhang et al. [34]	Sprague-Dawley rat.	Resveratrol	Decrease the cell apoptosis, mitochondrial dysfunction and radical oxygen species generation both in vitro and in vivo experiments, and normalised retinal morphology in vivo.
2019	Yang et al. [35]	Adult Fischer F344 rat.	Green tea extract	Higher number of surviving retinal ganglion cells, less apoptotic RGCs, smaller constricted pupil area and reduction of inflammation and apoptosis-related protein expressions.
2019	Wu et al. [36]	Spray-Dawley rat.	Szeto-Schiller peptide 31 (SS-31)	Neuroprotective effects by reducing oxidative stress and inhibiting mitochondrial-mediated apoptosis.
2019	Angel Aillegas et al. [37]	New Zealand rabbit.	Ascorbyl Laurate (ASC12)	Increment in kinetic antioxidant capacity in the aqueous humour of normotensive and hypertensive rabbits.
2020	Cao et al. [38]	C57BL/6JJcl mice.	Resveratrol (RSV)	Strong cytoprotective action against cell death through multiple pathways under high IOP.
2020	Chou et al. [39]	DBA/2J mice.	Vitamin B	Preservation of retinal ganglion cell function, density and mitochondria.
2020	Tanaka-Gonome et al. [40]	GLAST-/-and C57BL/6J mice.	Astaxanthin (AST)	Attenuation of the thinning of ganglion cell complex in a model of normal tension glaucoma.

**Table 2:** Summary of antioxidant supplementation in clinical trials (chronological order).

Year of Publication	Author	No. of Participants/Treatment	Antioxidant Substance	Results
1999	Cellini et al. [41]	40 ocular hypertensive patients (2 groups: treated versus placebo)	Omega-3 polyunsaturated fatty acids	Improvement mean defect (MD)with blue7yellow perimetry
2003	Quaranta et al. [42]	27 patients received 40 mg of GBE, taken orally, three times a day for 4 weeks, followed by an 8-week washout period, then 4 weeks of placebo treatment. Other patients underwent the same regimen, but took the placebo first and the GBE in the end.	Ginkgo biloba (GBE)	After treatment with GBE, a significant improvement in the indices of the visual fields. There were not found changes significant in the intraocular pressure, the blood pressure or heart rate after the placebo treatment or GBE
2009	Falsini et al. [43]	18 patients with hypertension and 18 patients with glaucoma (OAG) were assigned to take placebo or epigallocatechin gallate (EGCG).	Gallate of epigalocatequina (EGCG)	Improvement of pattern electroretinogram (PERG’) in patients with open angle glaucoma but not in ocular hypertension. However, the perimetry automated standard showed no significant changes after EGCG or placebo
2011	Park et al., 2011. [44]	30 NTG patients (2 groups: treated versus placebo)	Ginkgo biloba extract	Increased flow retinal blood peripapillary
2012	Shim et al. [45]	302 patients, were divided into 3 groups, group 1 treated with Antianciokines, group 2 with GBE and group 3 were not treated (control group).	Ginkgo biloba extract (GBE) Antianciokines	After treatment with anthocyanins, the Average BCVA for all eyes got better from 0.16 (±0.34) a 0.11 (±0.18) units logMAR. After treatment with GBE, the deviation half of HVF improved from −5.25 (±6.13) to −4.31 (±5.60)
2012	Ohguro et al. [46]	39 patients were divided into two groups, group 1 treated with blackcurrant (BCA) and group 2 treated with placebo.	Grosellanegra (BCA)	After administration BCAC, the flow ocular blood during the period of observation of 24 months increased compared to patients treated with placebo. However, significant changes were not observed in the conditions systemic and ocular, including IOP during the 24 month period
2012	Vetrugno et al. [47]	97 patients, 52 in the treatment group and 45 in the control group. Patients were treated with a dietary supplement consisting of forskolin and rutin in addition to their pharmacological treatment.	Forskolina Rutin	All patients in the treatment group showed a further decrease of 10% (*P* < 0.01) of PIO. On the contrary, IOP values in the control group were stable from the beginning to the end of the treatment period.
2013	Ohguro et al. [48]	21 patients, 12 were treated with BCAC and 9 were treated with placebo	Grosella negra (BCAC)	There was a decrease statistically significant in IOP mean at 2 weeks and 4 weeks, test paired) from the start in healthy treated subjects with BCA. However, this was not observed in the group treated with placebo. Oral administration of BCAC can induce a decrease beneficial in IOP levels in healthy subjects as well as in patients with glaucoma
2013	Egorov et al. [49]	94 patients were divided into 3 groups: 50 patients received combined therapy with mexidol 100 mg and picamilon 150 mg, 22 patients received combined therapy with exidol 300 mg and picamilon 150 mg, 22 patients received only picamilon 150 mg	Mexidol Picamilon	Improvement was recorded in visual acuity, indications perimeter, electrophysiology and increase in the speed of blood flow
2013	Galbis-Estrada et al. [50]		Essential polyunsaturated fatty acids and antioxidants (Brusysec^®^)	Reduction of oxidative stress biomarkers (vascular endothelial growth factor, tumour necrosis factor α,interleukin-4, interleukin-6) subjective improvement of dry eye symptoms
2014	Bonyadi Jabbarpoor et al. [51]	34 angle glaucoma patients, 17 to receive 30 mg/day of aqueous saffron extract by mouth and 17 others received placebo treatment as a supplement of timolol and dorzolamide	Saffron extract	After three weeks of treatment, IOP decreased significantly to 10.9 ± 3.3 mmHg in the group of saffron in comparison with 13.5 ± 2.3 mmHg in the group at the end of the washing period, the IOP was 12.9 ± 3.0 in the saffron group versus 14.2 ± 2.0 mmHg in the control group (*p* = 0.175)
2014	*Guo et al.* [52]	35 NTG patients (2 groups: treated versus placebo)	Ginkgo biloba extract	No effect on automated perimetry or sensitivity to contrast
2015	*García-Medina et al.* [53]	35 patients NTG Oral supplementation versus placebo)	Two antioxidant formulas based on AREDS (antioxidants + minerals) with/without FA-3 PUFA	There are no differences significant among global indices perimeter, the RNFL peripapillary or the GCC macular at the beginning and at the end of the follow-up
2016	Mutolo et al. [54]		Complex composed of homotaurine, Coleus forskohlii root extract, Lcarnosine, folic acid, vitamins B1, B2, B6 and magnesium	We observe in patients treated a decrease in significant additional of the IOP and an improvement from the PERG amplitude to 6, 9, and 12 months, and sensitivity foveola 12 months
2018	Harris et al. [55]	45 patients treated, a group with the antioxidant and the other with placebo	Ginkgo biloba extract	Improvement in blood flow, a less vascular resistance in retina
2018	Romeo Villadoniga et al. [56]	47 patients (23 treated with DHA and 24 untreated	Docosahexaenoico acid (DHA)	In the DHA group, the PIO, the content of erythrocytes increased by the DHA group and TAC levels increased compared to the untreated group
2019	Ozates et al. [57]	64 patients (divided in two groups one treated with coenzyme Q10 and vitamin E and the other no treaty	Coenzyme Q10 Vitamin E	Mood levels lower watery in the group that was treated, same as one more level low superoxide dismutase. There were not significant changes observed in the level of malondialdehyde
2019	Quaranta et al. [58]	They will select a total of 612 patients	Coenzyme Q10 and Vitamin E	Improvement the already existing visual field damage in these patients. They achieved an MD reduction of 11.40 + 3.27 decibels (dB) compared to the 8.78 + 2.56 dB reduction in patients treated with placebo (*p* = 0.0001)
2020	Sanz-González et al. [59]	30 patients, which were divided in two groups, one group of patients with glaucoma and a group healthy patients	R-alpha lipoic acid, taurine, vitamins C and E, lutein, zeaxanthin, zinc, copper and acid docosahexaenoic	After 6 months of supplementation, there was a significant increase in the total antioxidant status in plasma along with a parallel decrease in the group POAG. Malondialdehyde also decreased. Schirmer test improved (20–30%) and the subjective signs/symptoms of dry eye decreased notably in the POAG group in front of control group
